# Aurora-A kinase is differentially expressed in the nucleus and cytoplasm in normal Müllerian epithelium and benign, borderline and malignant serous ovarian neoplasms

**DOI:** 10.1186/s13000-021-01158-4

**Published:** 2021-10-27

**Authors:** Khaled J. Alkhateeb, Jason E. Crane, Müge Sak, Caitlin J. Jorgensen, James P. O’Donnell, Cory T. Zumbar, Jason A. Wozniak, Clarence R. Salazar, Anil V. Parwani, Norman L. Lehman

**Affiliations:** 1 Department of Pathology and Laboratory Medicine, The University of Louisville, Louisville, KY 40202 USA; 2 Department of Pathology and Laboratory Medicine, Henry Ford Hospital Detroit, Detroit, MI 48202 USA; 3 Department of Biochemistry and Molecular Genetics, The University of Louisville, Louisville, KY 40202 USA; 4 Department of Pathology, Ohio State University, Columbus, OH 43210 USA; 5 Department of Pathology, Wayne State University, Detroit, MI 48201 USA; 6 The Brown Cancer Center, The University of Louisville, Louisville, KY 40202 USA

**Keywords:** Ovarian, Aurora-a, Nuclear, Cytoplasmic, Differential localization, Immunohistochemistry

## Abstract

**Background:**

Aurora-A kinase is important for cellular proliferation and is implicated in the tumorigenesis of several malignancies, including of the ovary. Information regarding the expression patterns of Aurora-A in normal Müllerian epithelium as well as benign, borderline and malignant epithelial ovarian neoplasms is limited.

**Methods:**

We investigated Aurora-A expression by immunohistochemistry in 15 benign, 19 borderline and 17 malignant ovarian serous tumors, and 16 benign, 8 borderline, and 2 malignant ovarian mucinous tumors. Twelve fimbriae from seven patients served as normal Müllerian epithelium controls. We also examined Aurora-A protein expression by western blot in normal fimbriae and tumor specimens.

**Results:**

All normal fimbriae (*n* = 12) showed nuclear but not cytoplasmic Aurora-A immunoreactivity by immunohistochemistry. Benign ovarian tumors also showed strong nuclear Aurora-A immunoreactivity. Forty-eight percent (13/27) of borderline tumors demonstrated nuclear Aurora-A immunoreactivity, while the remainder (52%, 14/27) lacked Aurora-A staining. Nuclear Aurora-A immunoreactivity was absent in all malignant serous tumors, however, 47% (8/17) demonstrated perinuclear cytoplasmic staining. These results were statistically significant when tumor class (benign/borderline/malignant) was compared to immunoreactivity localization or intensity (Fisher Exact Test, *p* < 0.01). Western blot analysis confirmed the greater nuclear Aurora-A expression in control Müllerian epithelium compared to borderline and malignant tumors.

**Conclusion:**

Aurora-A kinase is differentially expressed across normal Müllerian epithelium, benign and borderline serous and mucinous ovarian epithelial neoplasms and malignant serous ovarian tumors., with nuclear expression of unphosphorylated Aurora-A being present in normal and benign neoplastic epithelium, and lost in malignant serous neoplasms. Further studies of the possible biological and clinical implications of the loss of nuclear Aurora-A expression in ovarian tumors, and its role in ovarian carcinogenesis are warranted.

**Supplementary Information:**

The online version contains supplementary material available at 10.1186/s13000-021-01158-4.

## Background

Aurora-A is a serine-threonine kinase involved in cell cycle progression and mitosis [1, 2]. Aurora-A misexpression may lead to mitotic errors and genomic instability [2]. Its overexpression has been implicated in the tumorigenesis of several malignant neoplasms, including hematolymphoid lesions [3], gliomas [4, 5], medulloblastomas [6], and carcinomas of the breast [7], gastrointestinal tract [8, 9] and ovary [10].

In normal tissues, Aurora-A directly interacts and co-localizes with the nuclear pore complex in a transient manner at the metaphase-anaphase transition during mitosis. Aberrance of nuclear pore complex components prevents Aurora-A translocation into the nucleus and has been shown to cause polyploidy and mitotic catastrophe, potentially increasing the risk of chromosomal translocations and mutations during early stages of cancer development [11, 12].

In ovarian carcinoma, Aurora-A mediates cell migration and adhesion [13]. Inhibition of Aurora-A prevents the epithelial-to-mesenchymal transition, which is correlated with more aggressive tumor progression and metastasis [14]. Aurora-A promotes cell cycle progression and genomic instability through repression of p21, pRb, and BRCA2 [15], and overexpression has been associated with tumor progression and poor prognosis [16]. In the current study, we evaluated the differential nuclear and cytoplasmic expression of Aurora-A in benign, borderline and malignant serous and mucinous ovarian tumors using immunohistochemical and western blot analyses.

## Methods

### Tumors and control Normal tissue

The use of human tissues was approved by the Henry Ford Health System Institutional Review Board. Using the search terms “serous cystadenoma, serous borderline tumor, serous carcinoma, mucinous cystadenoma, mucinous borderline tumor, and mucinous carcinoma” we identified 84 ovarian neoplasms in our pathology case files. Cases signed out as mixed serous and mucinous neoplasms were excluded from the study. Seventy-seven cases remained: 15 benign serous tumors, 19 borderline serous tumors, 17 malignant serous tumors (including 16 high-grade serous carcinomas and 1 low-grade serous carcinoma), 16 benign mucinous tumors, 8 borderline mucinous tumors and 2 primary ovarian mucinous adenocarcinomas. Tumor diagnoses were confirmed by consensus review by three pathologists (KJA, JEC, NLL). Additionally, 12 fimbriae from 7 patients were included as normal tissue controls.

### Immunohistochemistry

Four-micron thick paraffin-embedded sections were incubated with anti-human Aurora-A antibodies (Dako North America, Inc., 1:500 dilution). Slides received heat induced epitope retrieval (HIER) using Envision FLEX Target Retrieval Solution Low pH, Citrate Buffer pH 6.1 (TRL). HIER was performed in a DAKO PT LINK Chamber. In a DAKO LINK Autostainer, endogenous peroxide was blocked using 3% hydrogen peroxide for 5 min. The primary antibody was incubated for 20 min. Visualization was achieved via a 15 min incubation of FLEX + Rabbit Linker, followed by a 20 min incubation of FLEX HRP (Dextran coupled with peroxidase and goat secondary antibody against rabbit and mouse immunoglobulins), and a 10 min incubation with DAB Chromogen (3,3′-diaminobenzidine tetrahydrochloride). TRIS buffer washes were performed between each incubation. Slides were counterstained with Mayer’s Hematoxylin for 5 min and washed for 15 min in tap water. Interpretation of staining pattern and intensity was performed independently by two of the study pathologists (JEC, NLL) and a consensus was then reached. Positive staining was defined as greater than or equal 5% of tumor cell immunoreactivity. The immunoreactivity localization was recorded as absent, cytoplasmic, or nuclear immunoreactivity. The intensity of immunolabeling was scored on a scale of 0 to 3 (0, negative, 1, weak, 2, moderate, 3, strong).

### Tissue lysates and Western blotting

Samples of normal fimbriae and ovarian tumors were obtained from fresh surgical specimens, snap frozen in liquid nitrogen and stored in − 80 degrees Celsius. Frozen tissue was homogenized on ice and processed into nuclear and cytosolic fractions as previously described [17]. Protease inhibitors (aprotinin (Sigma, St. Louis, MO), leupeptin, pepstatin A, chymostatin, and AEBSF (MP Biomedicals, Solon, OH)) and 1 μM DTT were added to all lysis buffers. Protein concentration was determined by the Pierce BCA method (Thermo Fisher Scientific, Waltham, MA). Nuclear and cytosolic fractions (20 μg total protein per lane) were electrophoresed on 10% polyacrylamide gels and electrotransferred to PVDF membranes (Millipore, Billerica, MA). Blocking was performed with 4% dried milk in TBST. Membranes were incubated with anti-human Aurora-A antibodies (Abcam, ab13824, 1:500), anti-human phospho- Thr^288^-Aurora-A (Abcam, ab58494, 1:500) or anti-human β-actin (Sigma, A2228, 1:5000) antibodies, followed by incubation with goat anti-mouse IgG-HRP (Santa Cruz Biotechnologies, Santa Cruz, CA) secondary antibody. Blots were developed with Pierce ECL (Thermo Fisher Scientific, Waltham, MA) and exposed to X-ray film.

### Statistical analysis

Using VassarStats online statistical software (http://www.vassarstats.net/), Fisher exact test was used to determine the significance of differences in Aurora-A immunohistochemical stain localization and intensity between benign, borderline and malignant ovarian neoplasms. A *p*-value of less than 0.05 was considered statistically significant.

## Results

### Immunohistochemistry

**Fig. 1:**
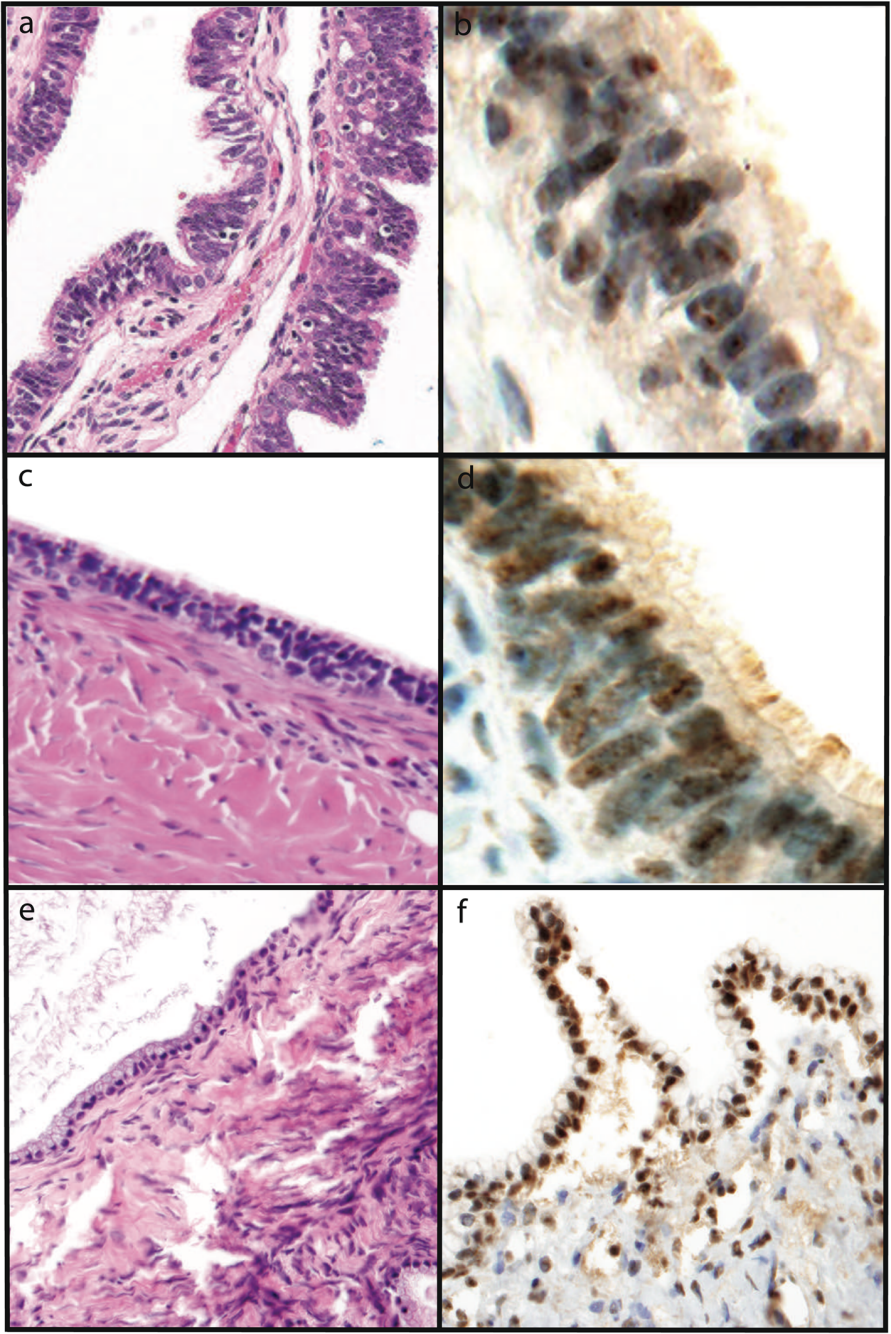
H&E stains and Aurora-A immunohistochemistry in normal tissue and benign neoplasms. Hematoxylin and eosin **(**H&E) stains and Aurora-A nuclear immunoreactivity in control normal Müllerian epithelium (a, 200x original magnification, and b, 400x original magnification) and benign serous (c, 200x original magnification, and d, 400x original magnification) and mucinous cystadenomas (e and f, 200x original magnification)

**Fig. 2:**
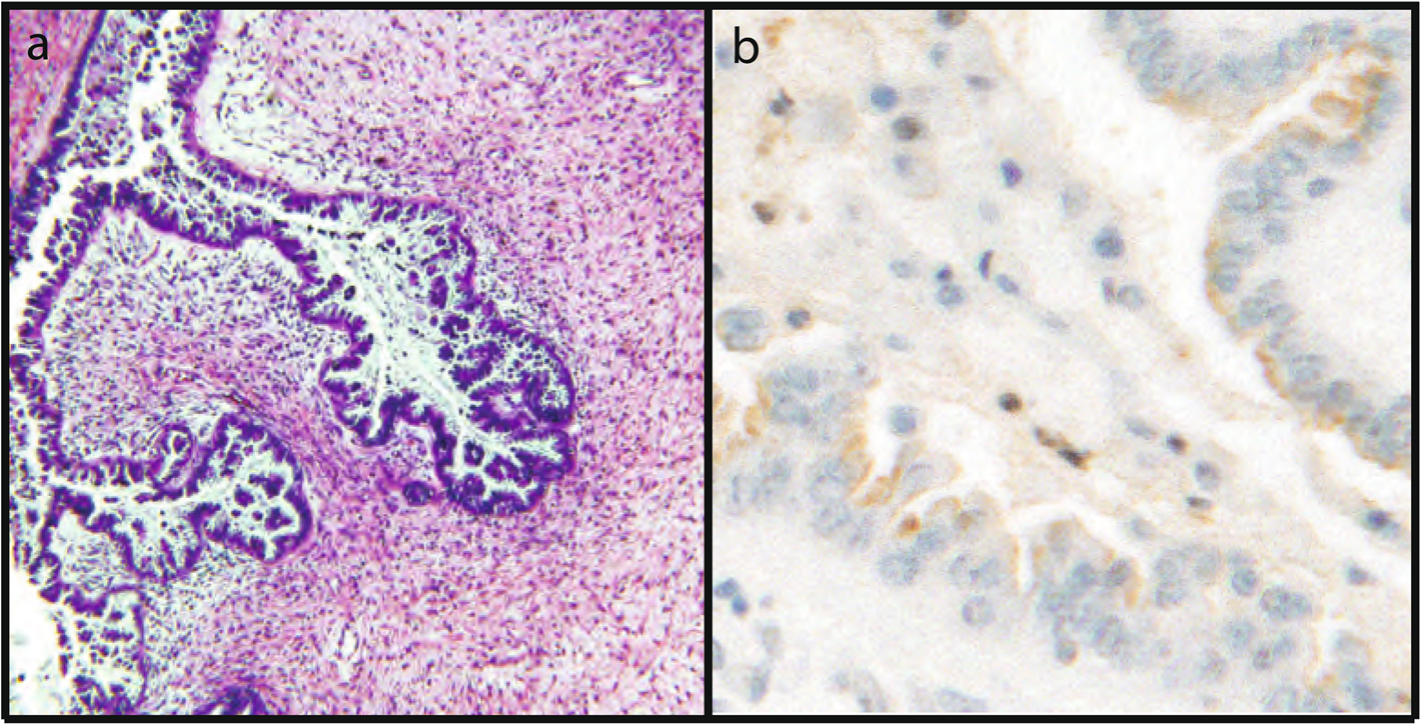
H&E and absence of Aurora-A immunoreactivity in a borderline serous tumor (a, 100x original magnification, b 400x original magnification)

**Fig. 3:**
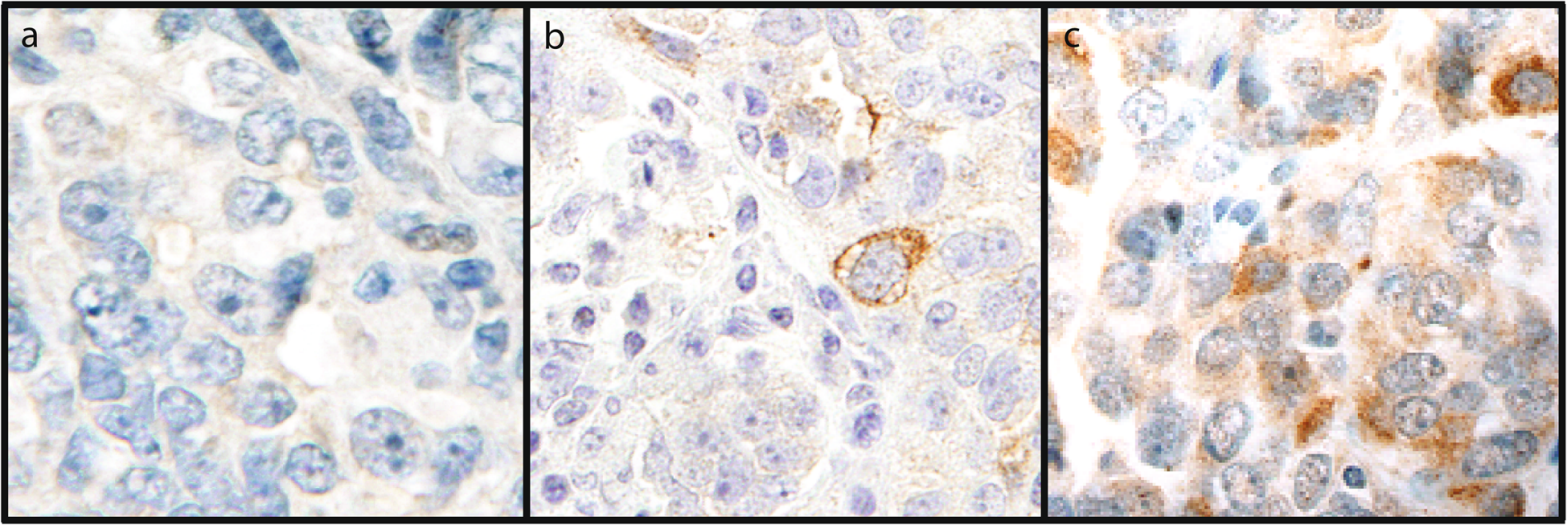
Aurora-A immunoreactivity in high-grade serous carcinoma. High-grade serous carcinomas with negative (a), weak (b) and strong (c) cytoplasmic Aurora-A immunoreactivity (400x original magnification)

There were 26 cases (34%) that were negative for Aurora-A by immunohistochemistry (IHC), 8 cases (10%) with cytoplasmic immunoreactivity, and 43 cases (56%) with nuclear immunoreactivity. All 12 normal fimbriae showed nuclear Aurora-A immunoreactivity. Almost all benign serous and mucinous tumors showed moderate to strong nuclear staining (Fig. 1). Weak nonspecific cytoplasmic “blush-like” staining was present in some of the normal control and benign cases; however, no convincing perinuclear cytoplasmic staining was demonstrated. Nuclear immunoreactivity was present in 42 and 62.5% of serous and mucinous borderline tumors, respectively. The remaining borderline tumors (52%) lacked any Aurora-A immunoreactivity (Fig. 2). In contrast, nuclear immunoreactivity was absent in all 17 malignant serous tumors. One of two mucinous carcinomas showed weak nuclear staining, however data from only two samples is insufficient to draw any conclusions from. Forty-two percent (8/19) of the malignant tumors demonstrated perinuclear cytoplasmic immunoreactivity for Aurora-A (Table 1), ranging from weak to strong (Table 2) (Fig. 3). Using Fisher exact test, the results were found to be highly statistically significant when tumor class (benign/borderline/malignant) was analyzed against immunoreactivity pattern (absent/cytoplasmic/nuclear) or intensity (weak, moderate, strong) (*p* < 0.01). The only exception was when comparing Aurora-A staining intensity between all serous and mucinous borderline and malignant tumors. (Table 3).

**Table 1:** Aurora-A staining localization for each tumor type

Tissue	Type	Absent	Cytoplasmic	Nuclear	Total
Fimbriae	Control	0	0	12	12
Serous Tumors	Benign	1	0	14	15
	Borderline	11	0	8	19
	Malignant	9	8	0	17
	Total	21	8	22	51
Mucinous Tumors	Benign	1	0	15	16
	Borderline	3	0	5	8
	Malignant	1	0	1	2
	Total	5	0	21	26
All Tumors	Benign	2	0	29	31
	Borderline	14	0	13	27
	Malignant	10	8	1	19
	Total	26	8	43	77

**Table 2:** Aurora-A staining intensity for each tumor type

Tissue	Type	Absent	Weak (1)	Moderate (2)	Strong (3)	Total
Fimbriae	Control	0	2	5	5	12
Serous Tumors	Benign	1	1	5	8	15
	Borderline	11	6	2	0	19
	Malignant	9	5	1	2	17
	Total	21	12	8	10	51
Mucinous Tumors	Benign	1	1	8	6	16
	Borderline	3	2	1	2	8
	Malignant	1	1	0	0	2
	Total	5	4	9	8	26
All Tumors	Benign	2	2	13	14	31
	Borderline	14	8	3	2	27
	Malignant	10	6	1	2	19
	Total	26	16	17	18	77

**Table 3:** Results of Fisher’s Exact Test for comparisons of Aurora-A immunoreactivity localization and intensity between different tumor categories

Tumor Categories	***P*** value (Fishers Exact Test)
Aurora-A Localization in all Benign vs. Malignant Tumors	9.340E-11
Aurora-A Localization in all Borderline vs. Malignant Tumors	0.00002
Aurora-A Localization in all Benign vs. Borderline Tumors	0.00022
Aurora-A Intensity in all Benign vs. Malignant Tumors	0.000005
Aurora-A Intensity in all Borderline vs. Malignant Tumors	0.9
Aurora-A Intensity in all Benign vs. Borderline Tumors	0.0000014
Aurora-A Localization in Benign vs. Malignant Serous Tumors	3.181E-8
Aurora-A Localization in Borderline vs. Malignant Serous Tumors	0.00006
Aurora-A Localization in Benign vs. Borderline Serous Tumors	0.003
Aurora-A Intensity in Benign vs. Malignant Serous Tumors	0.001
Aurora-A Intensity in Borderline vs. Malignant Serous Tumors	0.62
Aurora-A Intensity in Benign vs. Borderline Serous Tumors	0.00002

### Western blotting

**Fig. 4:**
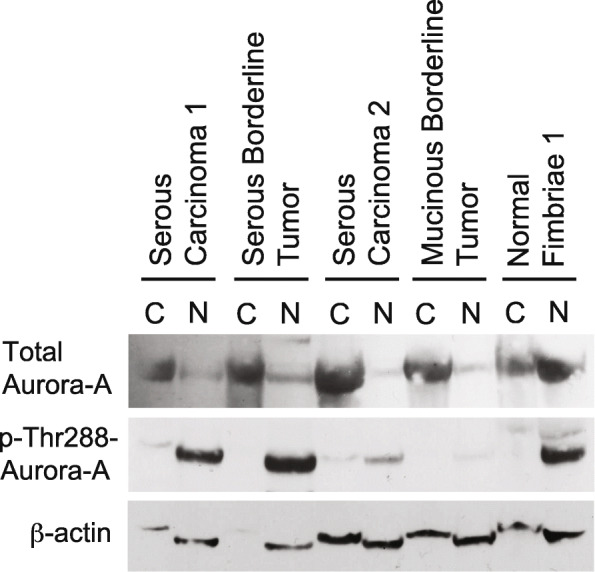
Western blotting for Aurora-A and phospho-Thr^288^-Aurora-A. Western blotting for the localization of total Aurora-A and phospho-Thr^288^-Aurora-A in the cytoplasmic and nuclear fractions of tumor lysates. Loading of total protein for the cytoplasmic fraction of the serous borderline tumor example appears lower as indicated by low β-actin, however this sample still shows higher cytoplasmic total Aurora-A. The latter is confirmed on another western blot utilizing this sample depicted in Supplemental Fig. 1

Western blotting for Aurora-A protein confirmed its predominantly cytoplasmic localization in malignant serous carcinomas, as well as in a borderline serous tumor, and less so a borderline mucinous tumor. In contrast, a near equal nuclear and cytoplasmic distribution of Aurora-A was observed in normal fimbriae (Fig. 4, Supplemental Fig. 1). Unlike total Aurora-A protein, phospho-Thr^288^-Aurora-A was heavily concentrated in the nuclear compartment of benign, borderline, and malignant serous ovarian tumors, as a whole. (Fig. 4, Supplemental Fig. 1).

## Discussion

Because of the complex structure and natural history of the adult ovary it is often difficult to demonstrate normal benign ovarian epithelium as a control for immunohistochemical studies and essentially impossible to isolate sufficient amounts for western blotting. Benign Müllerian epithelium, most readily available in normal fallopian tube fimbriae, shows gene expression patterns similar to serous ovarian tumors [18] and is a good normal control for serous tumors, at least some of which arise in the distal fallopian tube [19, 20].

Aurora-A expression has been reported to be an independent prognostic factor for progression-free survival in ovarian carcinoma [21]. One study correlated nuclear and cytoplasmic Aurora-A overexpression in ovarian serous carcinoma with shorter survival, high grade, high proliferation index, and aberrant p53 expression [22]. Interestingly, that study also found that only cytoplasmic Aurora-A expression was associated with tumor cell aneuploidy, which was a strong predictor of poor outcome. Yet the biology of Aurora-A is complex, and it may also possibly function as a tumor suppressor [4].

We found that benign fimbriae had the highest nuclear to cytoplasmic ratio of total Aurora-A based on western blotting. Benign serous and mucinous ovarian tumors also showed strong nuclear immunoreactivity by immunohistochemistry. Borderline tumors tended to show nuclear immunoreactivity like benign tumors, however it was generally weaker, and they sometimes lacked nuclear staining like malignant tumors. In contrast, none of the malignant serous tumors we examined demonstrated nuclear Aurora-A immunoreactivity. Unlike benign and borderline tumors, malignant serous tumors sometimes showed cytoplasmic immunoreactivity for Aurora-A. This is in line with previous work showing low Emi1 expression in the cytoplasm of neoplastic cells in some serous ovarian carcinomas through immunohistochemical analysis [23], as Emi1 protects Aurora-A from degradation by the anaphase promoting complex/cyclosome [24]. Notably, the differential localization of other mitotic spindle proteins in benign and malignant tissue has also been reported [11].

Although nuclear total Aurora-A expression was not detected in serous carcinoma by immunohistochemistry, nuclear phospho-Thr^288^-Aurora-A expression was identified in serous carcinomas by western blotting. It is possible that phospho-Thr^288^-Aurora-A is less efficiently recognized by the “total’ anti-Aurora-A antibody, is much less abundant compared to total Aurora-A, or both. Nevertheless, there appears to be decreased accumulation of total Aurora-A in the nucleus of serous ovarian carcinomas and often increased accumulation in the cytoplasm, where it is known to perform many of its pro-mitotic functions [25]. This finding was demonstrated by both IHC and western blotting. It must be remembered that negative immunohistochemistry does not mean the protein is absent from the cell, but that it is not detectable by this relatively insensitive method.

Phosphorylation of Aurora-A at Thr^288^ correlates with activation of its kinase activity. The nuclear and cytoplasmic localization of Aurora-A, however, does not appear to be dependent on its kinase activation [26]. Furthermore, Aurora-A may be able to perform functions inside the nucleus that are not related to its kinase domain, including possibly acting as a transcriptional coactivator [27]. This should not be surprising given that although there is a large degree of homology between the catalytic domains of all human Aurora proteins, they perform unique roles inside the cell for which their precise localization is a key factor [28]. Indeed, the functional differences between Aurora-A and Aurora-B are determined by their spatial compartmentalization [29]. This suggests that spatial regulation could be an important factor in the oncogenic role of Aurora-A. Increased cytoplasmic staining of Aurora-A in malignant cells may be due to increased Aurora-A transcription, thus overwhelming its nuclear transport and leading to cytoplasmic accumulation and decreased nuclear accumulation of unphosphorylated Aurora-A.

Negative nuclear Aurora-A expression in malignant and some borderline tumors may have potential implications in the biology of serous ovarian tumors. The fact that inhibition of Aurora-A has been found to synergistically enhance the cytotoxicity of taxanes [30–32] and carboplatin [33, 34], two of the most important chemotherapeutics for ovarian cancers [35], provides justification for further study of the possible roles of Aurora-A in the diagnosis and treatment of ovarian neoplasms.

## Conclusion

Aurora-A kinase is differentially expressed across normal Müllerian epithelium, benign and borderline serous and mucinous ovarian epithelial neoplasms and malignant serous ovarian tumors. Normal Müllerian epithelium as well as benign ovarian neoplasms show distinct nuclear expression of Aurora-A on both IHC and western blot, while malignant serous ovarian tumors demonstrate loss of nuclear expression, but showed perinuclear cytoplasmic immunoreactivity in approximately 50% of cases on IHC. Further studies are warranted in order to further understand the possible biological and clinical implications of the loss of non-phospho-Thr^288^-Aurora-A nuclear expression in ovarian tumors, and its role in ovarian carcinogenesis.

## Supplementary Information

**Additional file 1: Supplemental Figure 1.** Additional western blot of neoplasm samples used in Fig. 4 for total Aurora-A showing similar results (a). Separate western blots of normal fimbriae for total Aurora-A showing approximately equal nuclear and cytoplasmic accumulation.

## Data Availability

Not applicable
