# Supplementary figures and images for: Aurora-A kinase is differentially expressed in the nucleus and cytoplasm in normal Müllerian epithelium and benign, borderline and malignant serous ovarian neoplasms
Source: Diagn Pathol. 2021 Oct 27;16:98. doi: 10.1186/s13000-021-01158-4 (PMC8549328; doi:10.1186/s13000-021-01158-4)

Fig. S1

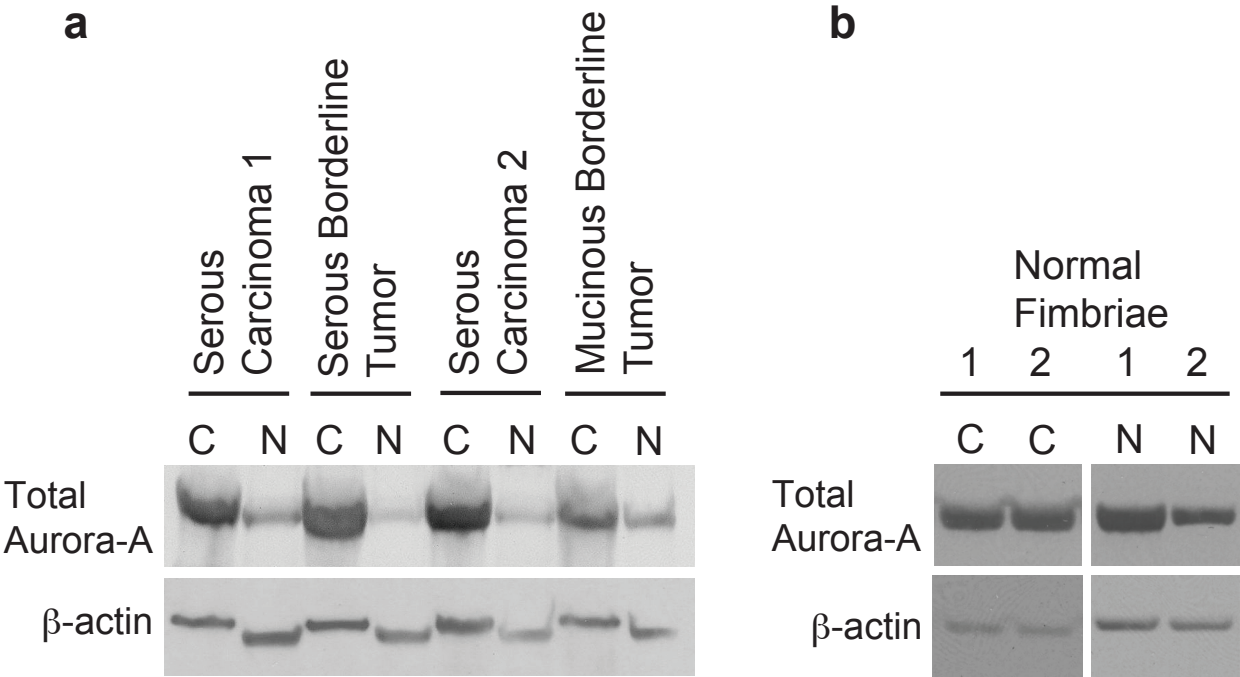

Supplement: Supplementary file 1 — Additional file 1: Supplemental Figure 1. Additional western blot of neoplasm samples used in Fig. 4 for total Aurora-A showing similar results (a). Separate western blots of normal fimbriae for total Aurora-A showing approximately equal nuclear and cytoplasmic accumulation. [file 13000_2021_1158_MOESM1_ESM.pdf]
